# Regulation of bile acids homeostasis: a feasible and versatile way to treat or diagnose liver disorders

**DOI:** 10.3389/fnut.2026.1797381

**Published:** 2026-04-14

**Authors:** Qian-Qian Wu, Le-Ying Gao, Hui-Yi Feng, Hao-Lin Liu, Hui Gao, Wei Peng, Nan Li

**Affiliations:** 1 School of Modern Chinese Medicine Industry, School of Pharmacy, Chengdu University of Traditional Chinese Medicine, Chengdu, China; 2 Chinese Medicine Germplasm Resources Innovation and Effective Uses Key Laboratory of Sichuan Province, Chengdu University of Traditional Chinese Medicine, Chengdu, China; 3 College of Pharmacy, Liaoning University of Traditional Chinese Medicine, Dalian, China

**Keywords:** BAs, cholestasis, cirrhosis, hepatocellular carcinoma, liver fibrosis, MASLD/MASH

## Abstract

Homeostatic imbalance of bile acids (BAs) is closely associated with the onset and progression of several liver diseases, including cholestasis, hepatic fibrosis, cirrhosis, hepatocellular carcinoma, and MASLD/MASH. In cholestasis, BAs imbalance induces cellular endoplasmic reticulum stress and mitochondrial dysfunction, which subsequently lead to hepatocellular and cholangiocellular death, exacerbating liver injury. Bile acid imbalance also activates hepatic stellate cells, promoting fibrosis and cirrhosis. During hepatocellular carcinoma development and progression, BAs imbalance favors the survival and proliferation of cancerous hepatocytes. Similarly, in MASLD/MASH, homeostatic BAs imbalance correlates significantly with disease severity. Additionally, BAs, through crosstalk with gut microbes, plays a key role in the development and progression of various liver diseases. This review focuses on each liver disease, summarizing the unique changes in BAs species and their distinct mechanisms of action, while exploring the complex role of BAs throughout the course of liver disease and their potential as diagnostic markers. Importantly, it highlights the therapeutic significance of regulating BA homeostasis in treating liver diseases.

## Introduction

Bile acids (BAs) are well known for their roles in lipid digestion and absorption, as well as functioning as signaling molecules that regulate glucose, lipid, and amino acid metabolism and maintain gut microbiota homeostasis (1, 2). In the liver, cholesterol is metabolized into BAs by 17 different enzymes (3). Approximately 98% of this BA metabolism is catalyzed in the final step by BA’s coenzyme A: amino acid N-acyltransferase, which typically conjugates glycine and taurine to the C24 position via an amide bond. N-acyltransferases are selectively expressed and predominantly enriched in the liver (4). Thus, the liver is the primary site for BAs conjugation with glycine and taurine, forming compounds such as taurocholic acid. BAs are classified as primary or secondary based on their origin. Primary BAs are synthesized directly in the liver from cholesterol and include cholic acid (CA), chenodeoxycholic acid (CDCA), and their conjugates with glycine or taurine (5). Secondary BAs are formed when primary BAs are secreted into the intestines and undergo 7α-dehydroxylation by intestinal microorganisms, mainly producing deoxycholic acid (DCA) and lithocholic acid (LCA), along with their glycine or taurine conjugates (6, 7).

There is a complex pathological crosstalk between the liver and BAs homeostasis rather than unidirectional regulation. Liver disease can induce BA imbalances, which in turn exacerbates liver injury, creating a vicious cycle. Therefore, this review summarizes the pathogenic roles and distinctive alterations of BAs in various liver diseases and discusses their complex diagnostic potential throughout the progression of liver disease, providing a theoretical basis for clinical treatment.

## BAs metabolism and negative feedback regulatory mechanisms

### Metabolic processes of BAs

Primary bile salts do not possess the membrane permeability of BAs and can only be transported into the bile by the bile salt export pump (BSEP, ABCB11). They can be stored in the gallbladder until released into the duodenum after food ingestion (8). The initial “gateway” reaction of bile salt metabolism by bacteria is mediated by bile salt hydrolase (BSH), which catalyzes the hydrolysis of conjugated bile salts to release primary free BAs (CA, CDCA) and amino acids (9).

Past studies have only identified the uncoupling activity of BSH. However, more recent research by Guzior et al. (10, 11)has demonstrated that BSH can also conjugate unconjugated BAs with the amino acids phenylalanine, leucine, and tyrosine to generate bacterial BAs amidate. Although available studies indicate that the maximum acyl transfer activity of BSH is only about 7% of its uncoupling activity, this process significantly expands the human BA pool. The antimicrobial activity of these gut microbe-mediated microbial conjugated BAs has been found to be more potent—though not exceeding that of free BAs—than classical taurocholic acid (TCA) and glycocholic acid (GCA). These novel microbially conjugated bile salts also activate primary host bile salt receptors, including the Farnesoid X receptor (FXR) and Takeda G protein-coupled receptor 5 (TGR5) (12). Meanwhile, unconjugated BAs are metabolized into secondary BAs through dehydroxylation, oxidation, and differential isomerization in the presence of gut microbes (13).

When unconjugated and conjugated BAs reach the terminal ileum, they are transported into enterocytes via the ileal BAs transporter (IBAT). IBAT interacts with recombinant human fatty acid-binding protein 6 and facilitates the translocation of BAs into the portal circulation through the Organic Solute Transporter alpha-beta complex, which are expressed on the basolateral membrane of enterocytes (1). The affinity of IBAT varies for different BAs; however, there remains controversy regarding its transport preference for conjugated versus unconjugated BAs (1, 14).

Conjugated BAs return to hepatocytes through a sodium-dependent mechanism, primarily mediated by the sodium/taurocholic acid cotransport polypeptide (NTCP) and the organic anion-transporting polypeptide (OATP). In contrast, unconjugated BAs are taken up by hepatocytes via a sodium-independent mechanism through OATP (6). Glycine- and taurine-conjugated BAs have been shown to be superior substrates for OATP uptake compared to unconjugated BAs (15). Overall, conjugated BAs appear to enter hepatocytes more readily.

### Negative feedback regulatory mechanisms of BAs

BAs mediate their endocrine effects by binding to and activating the nuclear receptor, FXR. According to Vaquero et al. (16), FXR expresses different isoforms in the liver and intestine. The most potent endogenous ligand for both the major isoform FXR1 (−/+) expressed in the liver and the major isoform FXR2 (−/+) in the intestine is CDCA, while DCA and LCA act as FXR antagonists in the presence of CDCA (17). FXR activation by BAs (18) regulates tissue-specific gene networks and coordinates synthesis and metabolism *in vivo*. In particular, through a negative feedback loop, activated FXR inhibits *de novo* hepatic BA synthesis by transactivating one of its major intestinal targets, the intestinal factor fibroblast growth factor 15/19 (FGF15 in mice and FGF19 in humans) (19). FGF15/19 is then secreted into the portal circulation and travels to the liver, where it binds to a heterodimeric co-receptor composed of fibroblast growth factor receptor 4 and Klotho-*β*. This binding initiates a phosphorylation signaling cascade that ultimately leads to CYP7A1(Cytochrome P450 Family 7 Subfamily A Member 1) inhibition, thereby suppressing *de novo* hepatic BAs synthesis (20).

Furthermore, the activation of FXR can increase the expression of the OSTα-OSTβ complex, leading to enhanced BA efflux within hepatocytes (21). Transcriptionally, BAs can influence miRNA expression. These miRNAs appear to be associated with the regulation of the BA homeostasis gene cluster. Among them, microRNA-34a exhibits a CDCA-dependent and strongly negative correlation with FGF19, the small heterodimer partner (NR0B2/SHP), OSTα-OSTβ complex, ATP-binding cassette subfamily G members 5 and 8, and solute carrier family 22 member 7 (SLC22A7) (22, 23). In addition to these mechanisms, recent studies have demonstrated that thyrotropin can inhibit hepatic BA synthesis via the SREBP-2 (sterol regulatory element-binding protein-2)/HNF-4α (hepatocyte nuclear factor 4α)/CYP7A1 signaling pathway (24).

## BAs and liver disease

### Biliary sludge

#### BAs induction promotes hepatic parenchymal cell death

**Figure 1:**
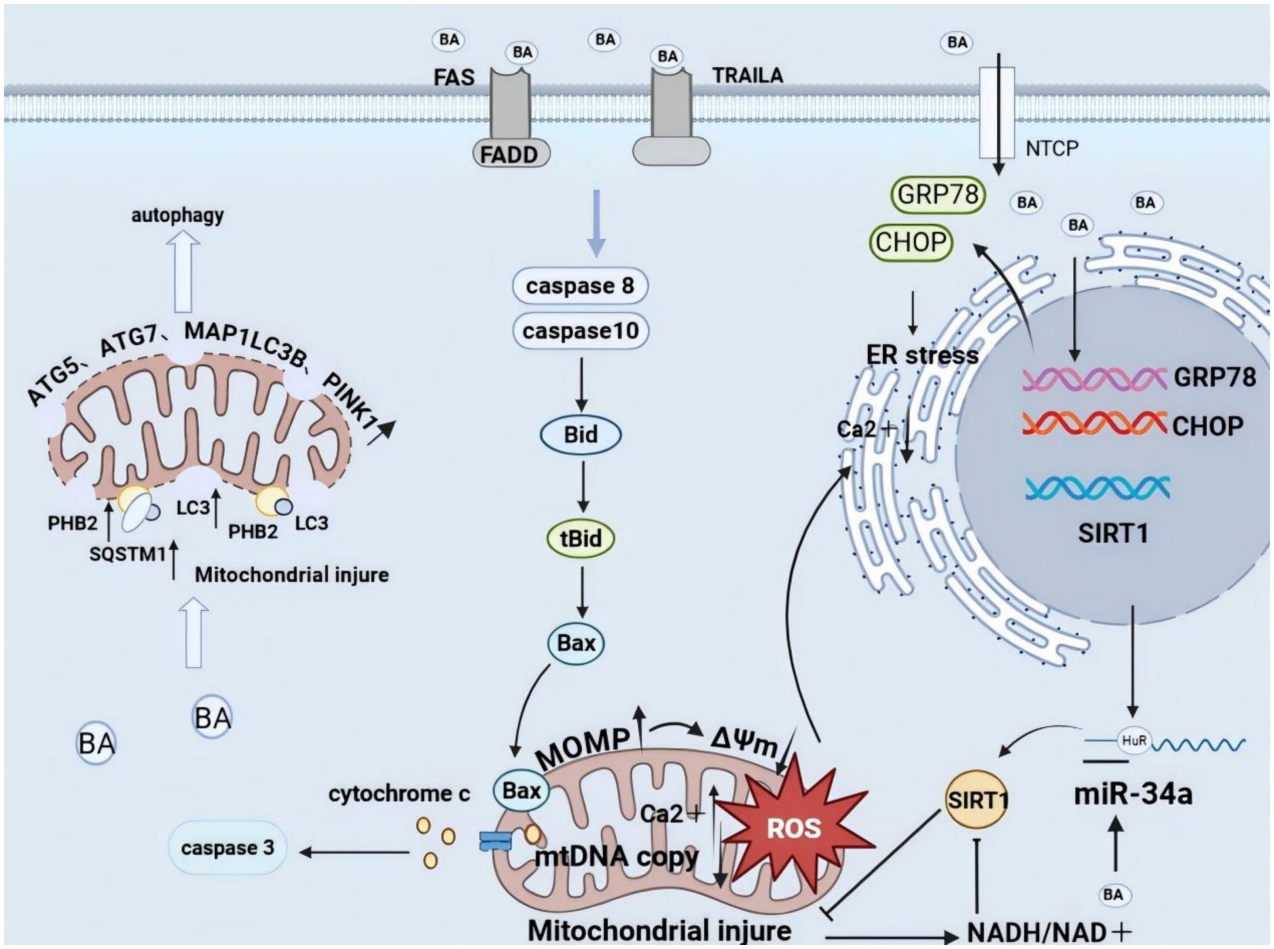
Hepatocyte apoptosis induced by hydrophobic bile acids. Hydrophobic BAs activate TNF-related apoptosis ligand receptor (TRAILR) and Fas death receptor signaling (FADD), activate the cleavage of Caspase 8 and Caspase 10 into tBid, activate the Bax translocation to mitochondria, and lead to the permeabilization of the outer membrane of the mitochondria (MOMP), which leads to the decrease of the mitochondrial membrane potential, the generation of ROS, the decrease of DNA copy number, and the release of cytochrome *c* into the cytoplasm, triggering a cascade reaction. The mitochondrial ROS surge and hydrophobic BAs induce the expression of the unfolded protein response (UPR) genes GRP78 and CHOP, which together lead to endoplasmic reticulum stress. Endoplasmic reticulum stress causes calcium iron overload. Hydrophobic BAs-mediated miR-34a inhibition downregulates SIRT1 mRNA expression, while mitochondrial dysfunction resulting in an elevated NADH/NAD^+^ ratio leads to inhibition of SIRT1 protein function. Hydrophobicbile salts bind the autophagosome membrane-associated protein LC3 to injured mitochondria by mediating the structural domain of the prohibitin 2 (PHB2) and LC3 interaction region. On the other hand, PHB2 forms a ternary protein complex with chelator 1 (SQSTM1) and LC3, leading to the loading of LC3 onto damaged mitochondria and causing mitochondrial autophagy (core autophagy-related protein: ATG5—autophagy related 5; ATG7—autophagy related 7; MAP1LC3B—microtubule-associated protein 1 light chain 3 beta; PINK1, PTEN induced kinase 1).

One of the core pathological features of cholestatic diseases is hepatocyte death caused by the excessive accumulation of hydrophobic BAs. *In vitro* studies have confirmed that BA-induced hepatocyte apoptosis primarily occurs through the mitochondrial-dependent pathway (25). Specifically, hydrophobic BAs can activate tumor necrosis factor-related apoptosis-inducing ligand receptors (TRAILRs) and Fas death receptors, initiating caspase-8 and caspase-10–mediated cascades. This leads to the cleavage of Bid into truncated Bid (tBid). tBid then cooperates with Bax to form pores in the mitochondrial outer membrane (MOM), increasing its permeability and ultimately resulting in the release of Cytochrome C into the cytoplasm, which activates downstream effector caspases (Figure 1) (26, 27).

#### Mechanisms involved in mitochondrial correlation during BAs-mediated hepatocyte death

Early studies predominantly used high concentrations of hydrophobic BAs, such as GCDCA, to treat hepatocyte models, revealing mechanisms by which these compounds induce mitochondrial dysfunction through multiple pathways. Hydrophobic BAs can directly trigger the opening of the mitochondrial permeability transition pore (mPTP), leading to membrane potential collapse and ATP depletion (28, 29). An imbalance in intracellular Ca^2+^ homeostasis plays a key role in this process, as the Ca^2+^ chelator BAPTA significantly attenuates BA-induced apoptosis (30). Furthermore, GCDCA treatment reduces mitochondrial DNA (mtDNA) copy number and induces oxidative stress (31). Rho cells lacking mtDNA exhibit enhanced resistance to GCDCA-induced cell death, suggesting that mtDNA damage is a critical component of BA toxicity (25).

Recent studies revealed that hydrophobic BAs may also indirectly impair mitochondria by affecting mitochondrial autophagy (e.g., through PHB2-mediated LC3 recruitment pathways) and potentially disrupting lipid and peroxisome autophagy processes (32, 33). Finally, BAs attenuate mitochondrial protective signaling pathways by suppressing SIRT1 expression and activity (34–36)—mechanisms involving JNK-mediated miR-34a upregulation (37) and an increased NADH/NAD^+^ ratio (38, 39)—thereby exacerbating cellular damage.

#### Mechanisms involved in BAs-mediated hepatocyte death involving the endoplasmic reticulum

The unfolded protein response (UPR) triggered by endoplasmic reticulum stress is a key feature of cholestatic liver injury (40). Hydrophobic BAs such as DCA and LCA induce the expression of UPR-associated genes GRP78 (41) and C/EBP homologous protein (CHOP) (42), while CHOP deficiency protects hepatocytes from BA-induced death both *in vivo* and *in vitro*. BA accumulation disrupts endoplasmic reticulum protein-folding homeostasis while elevating intracellular Ca^2+^ levels, triggering Ca^2+^-dependent apoptotic pathways (43); treatment with BAPTA significantly suppresses CDCA-induced GRP78 and CHOP upregulation and enhances cell survival (44). Disruption of endoplasmic reticulum Ca^2+^ homeostasis is partially attributable to reactive oxygen species (ROS) generated by mitochondria and the NADPH oxidase. The NADPH oxidase inhibitor DPI similarly suppresses GRP78 and TGF-β upregulation while enhancing cell survival (45). Endoplasmic reticulum stress and mitochondrial dysfunction form a vicious cycle via the Ca^2+^-ROS axis: endoplasmic reticulum stress causes cytoplasmic Ca^2+^ imbalance, which in turn damages the mitochondrial respiratory chain and increases ROS production. Conversely, ROS act on the endoplasmic reticulum, exacerbating protein-folding disorders. Mechanistically, TUDCA has been shown to inhibit the dissociation of GRP78 from Protein Kinase R-like Endoplasmic Reticulum Kinase (PERK), thereby attenuating PERK signaling and subsequent ER stress-mediated apoptosis (40). While TUDCA can inhibit downstream PERK pathways —specifically phospho-PERK, phospho-Eukaryotic Initiation Factor 2 Alpha, and CHOP—direct evidence for GRP78-PERK binding regulation remains limited to this single report (40, 46).

Most of the aforementioned studies employed high concentrations of hydrophobic BAs to treat cells, confirming their significant direct cytotoxic effects, which may represent the primary mechanism of liver injury in the late stages of cholestasis (47, 48). However, it should be noted that during the early stages of the disease or mild cholestasis, the concentration of accumulated BAs in patients is typically too low (approximately 5–15 μM) to induce significant cell necrosis or apoptosis through direct toxicity (49). Only when the disease progresses to a severe stage do BA levels reach 100–300 μM, at which point they become sufficient to trigger the aforementioned cytotoxic cascade. Therefore, the current consensus holds that in the early stages of the disease, disruption of BA homeostasis is more likely to affect cellular function by altering BA signaling networks rather than directly causing cell death. As feedback inhibition progressively diminishes, circulating BA levels continue to rise, gradually revealing their toxic effects and thereby driving disease progression.

#### BAs and itching

Research on the mechanism by which BAs induce pruritus has made significant breakthroughs in recent years. The most critical finding is the identification of MRGPRX4 as a BA receptor on human sensory neurons (50, 51). BAs can directly activate this receptor and mediate non-histamine-dependent pruritus signaling, providing a direct molecular basis for cholestatic pruritus. Concurrently, the ATX-LPA axis has been confirmed as another core pathway (52). Serum levels of the autocrine motility factor (ATX) are significantly elevated in patients with cholestatic pruritus, and ATX levels correlate with pruritus severity and treatment response (53). The role of truncated Bid (TGR5) faces considerable disagreement. Animal studies confirm TGR5 activation by BAs mediates pruritus via the TRPA1 channel (54, 55), but subsequent research reveals human TGR5 primarily expresses in satellite glial cells rather than sensory neurons—differing from mouse expression patterns (51). Clinical trials clearly demonstrate a significant increase in pruritus incidence during treatment with FXR agonists (e.g., OCA), though the mechanism remains unclear (56). This may involve indirect alteration of BA distribution through regulation of BA transporters (BSEP, NTCP, and MRP4). Nevertheless, the definitive anti-pruritic efficacy of IBAT inhibitors provides reverse evidence for BAs’ central role in pruritus pathogenesis (57). Future research should focus on identifying key pruritic BAs components and elucidating hierarchical relationships and species differences among pathways involving ATX-LPA, MRGPRX4, TGR5, and others.

#### Changes in BAs species to distinguish between different disease types or as a diagnostic criterion for diseases

Most of the studies have attempted to serve as disease markers by focusing on unique changes in species in BAs. For example, the differences in serum BAs profiles between the intrahepatic cholestasis of pregnancy (ICP) group and normal pregnant women were DCA, TDCA, TCA, GDCA, and GLCA (glycholithocholic acid) (58). However, ICP encompasses different subtypes, and comorbidities lead to significant variations in BA profiles. Bile acid patterns also differed between patients with genotyped cholestasis and those without a genetic diagnosis. Notably, the overall hydrophobicity indices of BAs were lower in patients with ABCB11 mutations, ABCB4 mutations, or undiagnosed cholestasis compared to healthy controls (59). A diagnostic panel comprising lithocholic acid (LCA), tauro-LCA, GLCA, and hyaluronic acid could distinguish the ABCB11 mutation cohort from healthy controls and patients with undiagnosed cholestasis and could also identify BSEP dysfunction in patients without ABCB11 mutations (60).

Secondary BAs were significantly lower in primary sclerosing cholangitis (PSC) and cholestatic controls than in non-cholestatic controls, whereas the BAs/goose deoxycholic acid ratio was significantly lower in PSC patients despite cholestasis, and the (CA + DCA)/(CDCA + LCA) ratio was significantly lower in PSC patients than in controls with or without cholestasis. The BA ratio in the bile of PSC patients showed a similar trend in serum (61). Thus, the difference in BAs is a promising indicator for differentiating PSC from cholestasis.

#### Bile acid levels predict disease future

Serum and fecal BA levels show great promise as non-invasive biomarkers for predicting disease progression. Multiple studies confirm serum BA levels correlate closely with fetal complications: each 1 μmol/L increase in BAs elevates fetal complication risk by 1–2%, and TBA ≥ 100 μmol/L significantly correlates with perinatal mortality, preterm birth, and meconium staining (62). However, complexity and controversy persist in this field—Dr. Shadi Rezai’s research revealed no direct linear relationship between ICP severity and spontaneous preterm birth, with the highest preterm birth rate observed in the mild group (63). Furthermore, the risk of stillbirth increased only in individuals with mildly elevated BAs (64), suggesting distinct mechanisms may underlie different outcomes. Notably, the predictive value of BAs has been cross-validated across multiple cholestatic disorders: in PFIC, pre-treatment CA + CDCA levels predict response to odevixibat therapy (AUC 0.76) (65), with serum BA reduction directly correlating to event-free survival (66). Collectively, these findings indicate that serum BAs serve as important observational predictors for ICP and other cholestatic diseases. However, further research is needed to elucidate the complex relationships between specific BA components and distinct clinical outcomes.

### Liver fibrosis and cirrhosis

#### Activation of hepatic stellate cells (HSCs) by BAs

For the activating effect of BAs on hepatic stellate cells, many believe that it is mainly related to the hydrophobicity of BAs, but taurocholic acid is an exception. TCA is a co-recognized promoter of hepatic fibrosis and promotes hepatic stellate cell activation under cholestatic conditions by regulating glutamate catabolism through sphingosine 1-phosphate receptor 2(S1PR2)/p38 mitogen-activated protein kinase (p38 MAPK)/yes-associated protein (YAP) signaling (67, 68). Salhab et al. first reported NTCP expression in human hepatic stellate cells and demonstrated that TCA uptake via NTCP promotes HSC activation and liver fibrosis progression, while NTCP inhibition attenuates fibrosis severity (69). Zheng et al. also observed dose-dependent activation of HSCs by TCA in a colorectal cancer liver metastasis model (70). However, van de Graaf et al. questioned this mechanism. Using the specific inhibitor Myrcludex B, they failed to reproduce NTCP-mediated TCA uptake in LX2 cells, suggesting existing evidence is insufficient to support the presence of functional NTCP on HSCs s (71). This controversy indicates that the actual role of the NTCP/HSCs axis in human liver fibrosis requires more rigorous experimental validation.

Treatment of HSCs with 500um of LCA and DCA revealed that the percentage of cells with high expression of *α*-smooth muscle actin (α-SMA) was significantly higher than that of the CA and CDCA-exposed groups (72). In contrast, another study found that 25, 50, and 100 μM CA upregulated early growth response protein 3 (EGR3) expression to activate Human hepatic stellate cells (LX-2), but DCA had no activating effect. The only difference between the two experiments was the concentration of BA, and it is possible that the concentration is the one that must be emphasized in the different signaling changes induced by BAs (73).

However, in a study using 100 μm DCA to treat mouse primary HSCs, it was found that DCA was able to promote hepatic stellate cell activation mediated by activation of nucleotide-binding oligomerization domain leucine-rich repeat containing receptors family pyrin domain-containing 3 (NLRP3) inflammatory vesicles, whereas CA and LCA did not lead to hepatic stellate cell activation in primary HSCs of mice, thus the different HSC cell lines also affected the action of BAs. In addition, LCA is thought to cause LX-2 apoptosis due to its excessive hydrophobicity (74, 75). In contrast, TUDCA, taurochenodeoxycholic acid (TCDCA), and ursodeoxycholic acid (UDCA) inhibit hepatic stellate cell activation.

**Figure 2:**
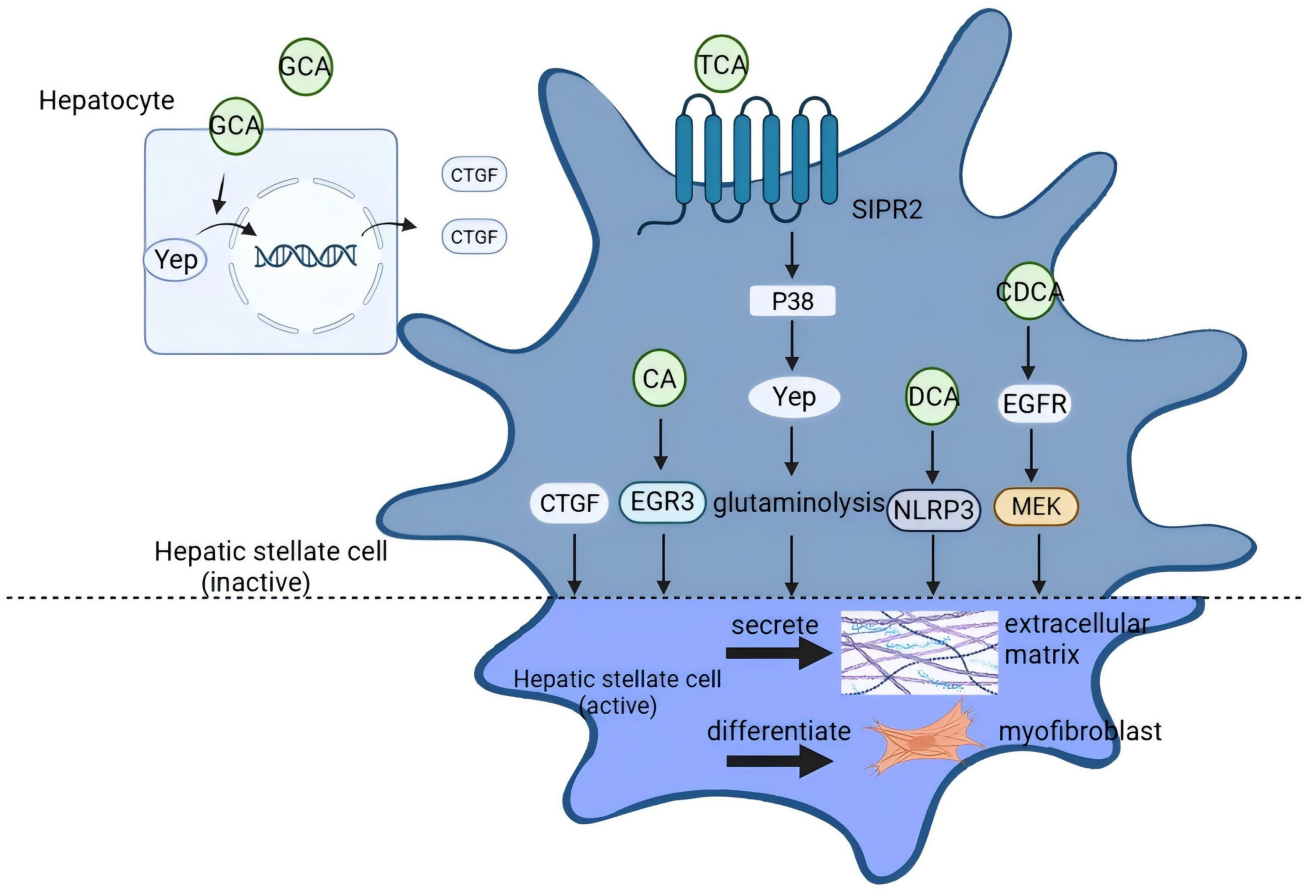
Signaling pathway of bile acid activation of hepatic stellate cells (HSC). Taurocholic acid (TCA) activates HSC by regulating glutamate catabolism through S1PR2/p38 MAPK/YAP signaling, and TCA also exerts its activating effect by entering HSC. CA upregulates early growth response protein 3 (EGR3) expression to activate hepatic stellate cells. CA is able to mediate the promotion of HSC activation through activation of inflammatory vesicles, NLRP3. GCA activates HSC through promotion of hepatocellular GCA activates HSC by inducing CTGF expression in hepatocytes by promoting nuclear translocation of YAP. CDCA induces epidermal growth factor receptor (EGFR)/MAP-ERK kinase (MEK)-dependent signaling cascade activation in hepatocytes.

In addition to direct action on HSC, some BAs are also able to promote HSC activation indirectly by affecting hepatocytes. GCA induces cellular communication network factor 2 (CTGF) expression in hepatocytes by promoting nuclear translocation of YAP, which activates HSCs and thus accelerates the process of liver fibrosis. However, GCA did not directly activate HSCs (76) but activation of the receptor FXR-SHP by BAs inhibited hepatic stellate cell activation (77, 78). Taken together, different BAs have different effects on hepatic stellate cells, and some of them may have either inhibitory or activating effects on hepatic stellate cells. For example, CDCA induced EGFR/MEK-dependent signaling cascade reaction to activate HSC (79) but activated FXR-SHP to inhibit the expression of collagen and TGF-β (77). Thus, under normal physiological conditions, BAs may just be offset from each other and from different signaling pathways activated by the same BAs. However, in pathological states, imbalances in BAs such as changes in concentration lead to the activation of hepatic stellate cells by BAs in a signaling pathway. Figure 2 shows the mechanism and key proteins involved in BA accumulation to activate HSCs.

#### Homeostatic changes in BAs in liver fibrosis and cirrhosis

During liver fibrosis progression, deoxycholic acid (DCA) exhibits prominent dynamic changes. The increased expression of microbial bile salt hydrolases, BA operons, and hydroxysteroid dehydrogenases—enzymes essential for DCA and its downstream metabolite synthesis—leads to elevated levels of several microbially derived BAs (including glycosides, 7-ketodeoxycholates, and dehydrocholates), which are closely associated with disease activity and fibrosis stage (80). Independent clinical trial cohorts have consistently demonstrated that DCA and its conjugates are associated with advanced fibrosis at enrollment. Notably, in placebo-treated patients, DCA levels decreased in those experiencing fibrosis regression and increased in those with fibrosis progression (80). DCA levels were further elevated upon decompensation in patients with compensated cirrhosis (80). The diagnostic value of DCA is further supported by a study in cystic fibrosis patients, where DCA distinguished non-cirrhotic liver disease from no liver disease with an area under the curve (AUC) of 0.867(95% Confidence Interval—CI, 0.822–1.000; *p* < 0.001) and 0.867 (95% CI, 0.731–1.000; *p* < 0.001) (81). However, this trend reverses in cirrhosis. Due to altered colonic microbiota and reduced bacterial 7α-dehydroxylase activity, DCA levels significantly decrease and are nearly absent in patients with advanced cirrhosis (82, 83). This opposite pattern of change—elevation during fibrosis and reduction in cirrhosis—positions DCA as a potential biomarker for distinguishing between fibrosis and cirrhosis (80, 82, 83).

Other BA species also demonstrate significant clinical correlations. In biliary atresia-induced liver fibrosis, glycochenodeoxycholic acid (GCDCA) shows a strong positive correlation with the fibrosis marker M2BPGi, whereas other BAs (GCA, TCA, and TCDCA) do not correlate with Child-Pugh scores, MELD scores, or M2BPGi (84). The pro-fibrotic and cytotoxic effects of GCDCA provide biological plausibility for this association (79). An integrated study combining human cohorts, clinical data, and animal models further revealed that taurocholate is significantly associated with F3 fibrosis (OR 8.56 × 10^−10^, FDR < 0.05), while taurochenodeoxycholate correlates with both early (F0 stage, OR 13.63, *p* < 0.05) and advanced disease stages (85). In cirrhotic patients and fibrotic mouse models, cholate (CA) accumulation is linked to SLC27A5 downregulation. SLC27A5 knockout induces spontaneous liver fibrosis at 24 months due to hepatic accumulation of unconjugated bile acids, particularly CA—an effect reversible by restoring SLC27A5 or reducing CA with the ASBT inhibitor A4250 (73). Additionally, increased levels of GCDCA, TCDCA, GCA, and TCA positively correlate with disease progression from Child-Pugh A to C and with MELD scores (86).

Beyond their role in staging cirrhosis, serum bile acid dynamics possess significant prognostic value. A prospective cohort study of 940 cirrhosis patients revealed that those in the highest quartile of baseline serum total bile acids had a markedly increased risk of progression to hepatocellular carcinoma (HCC) (adjusted HR = 3.69, 95% CI: 1.85–7.37) (87). Incorporating total BAs into clinical prediction models improved the c-statistic for predicting HCC progression within two years from 0.74 to 0.80 (*p* < 0.001) (87). These findings underscore that the serum bile acid profile undergoes substantial alterations during cirrhosis, with its dynamic changes reflecting progressive liver function deterioration (88). Consequently, the dynamic changes in serum BAs may serve as valuable biomarkers for cirrhosis staging and disease progression monitoring.

### Liver cancer

#### BAs facilitate chemotherapy resistance in hepatocellular carcinoma cells

Sun et al. (89) demonstrated that BAs generate an immunosuppressive tumor microenvironment favorable for tumor-initiating cell (TIC) expansion and tumor growth by activating FXR (Nr1h4) and skewing macrophage polarization. It is relatively undisputed that toxic BAs, especially GCDA, can reduce the chemosensitivity of HCC cells, but the exact mechanism remains somewhat controversial. The reduced chemosensitivity of HCC cells by GCDA in bile salts may occur through the up-regulation of the anti-apoptotic protein Myeloid cell leukemia-1 (Mcl-1)/Survivin/B-cell lymphoma-2 (Bcl-2) and is not related to GCDA-induced chemoresistance with signal transducer and activator of transcription 3 (STAT3) (90). Contrary to this, Wang et al. (91) showed that GCDA may activate STAT3 by phosphorylating it at Ser727 via the mitogen-activated protein kinase/extracellular signal-regulated kinase 1/2 (ERK1/2) pathway, and that GCDA-induced chemoresistance was eliminated when STAT3 phosphorylation was inhibited.

Epithelial mesenchymal transition (EMT) and cancer stem cell (CSC) acquisition are essential drivers of chemoresistance in HCC (92). Experiments by Shi et al. (93) showed that GCDC promotes chemoresistance in HCC by inducing stemness through the STAT3 pathway and may be a potential target for HCC chemotherapy. In addition, the mRNA and protein levels of E-calmodulin were down-regulated and those of waveform protein were up-regulated in GCDC-treated Huh7 and LM3 cells. These results suggest that GCDC for chemoresistance in hepatocellular carcinoma cells also promotes chemoresistance in HCC cells by conferring EMT phenotype and CSC properties.

**Figure 3:**
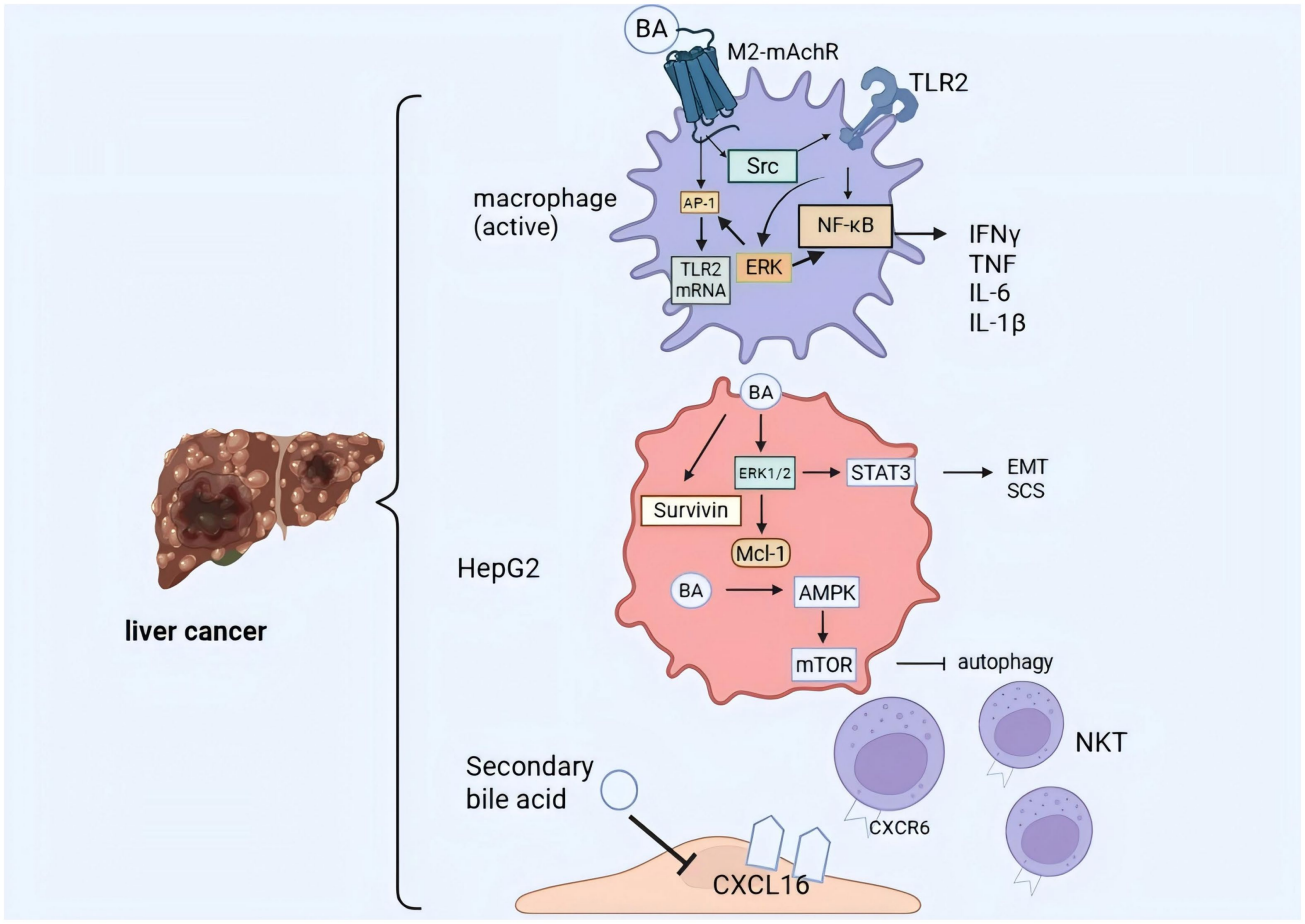
Mechanisms by which bile acids promote hepatocellular carcinoma development. Bile acids cause hepatocellular carcinoma cells to acquire an over-endowed EMT phenotype and CSC characteristics by up-regulating the anti-apoptotic proteins, Mcl-1/Survivin/Bcl-2, and by mediating the activation of STAT3 by ERK1/2. Bile acids mediate activating protein-1 (AP-1) via peripheral-muscarinic acetylcholine (M2-MAch) receptor leading to Toll-like Receptor 2 (TLR2) overexpression or Src leading to TLR2 translocation to the cell membrane. Activation of TLR2 leads to further increase in TLR2 expression via ERK and, more importantly, to activation of nuclear factor kappa B (NK-κB) and release of inflammatory factors. Secondary pro-bile acids were able to inhibit CXCL16 in LSEC cells, less NKT cell aggregation in the liver, and ultimately inhibit tumor growth.

GCDC enhances HCC cell invasion by mediating AMP-activated protein kinase (AMPK)/mechanistic target of rapamycin activation of autophagy (94). Autophagy plays a crucial role in the invasion of HCC cells upon exposure to BAs. Furthermore, accumulating evidence indicates that cytotoxic BAs promote HCC development through multiple mechanisms, including the expansion of tumor-initiating cells, modulation of the tumor microenvironment, suppression of chemosensitivity via STAT3 upregulation (contributing to therapeutic resistance), as well as the promotion of metastasis and recurrence (Figure 3). Therefore, targeting cytotoxic BAs represents a promising and multifaceted therapeutic strategy for HCC (95).

#### Unique changes in BAs profile due to hepatocellular carcinoma

Studies on the changes in BA profiles in patients with hepatocellular carcinoma have never ceased. The BA pool in hepatocellular carcinoma patients is enlarged compared with that in normal subjects, which may mediate inflammation (96) and suppress immunity to promote hepatocellular carcinoma formation. However, there has been controversy over the various types of BA changes. Elevated serum and urinary levels of conjugated BAs appeared to be associated with stage I HCC in a study by Chen et al. (97). In stage I HCC, a sudden increase in conjugated BAs (BAs) was observed, which gradually declined from stages II to IV. In another study, it was also found that in stage II and III HCC cases while GDCA, GCA, TCDCA were decreased (98). However, an increase in BAs in the pre-disease phase implies an increased risk of HCC (99). It can be simply surmised that conjugated BAs are in dynamic change during the disease process. From latency to stage I HCC, conjugated BA levels increase and begin to decrease in later stages. The ratios of the major secondary BA species (i.e., DCA/CA ratio, LCA/CDCA ratio, and UDCA/CDCA ratio) were significantly lower in HCC cases than in controls in a Singaporean study (100). Consistently, the serum ratio of secondary to primary BAs was significantly lower in the HCC group than in healthy controls (101). The percentage of secondary BAs in total BAs was significantly lower in both HCC groups (102). A major reason may be the reduced abundance of BSH-rich bacteria in the gut microbiota (103). Primary BAs stimulate increased expression of CXCL16 (CXC motif chemokine ligand 16) in liver sinusoidal endothelial cell (LSEC) cells, which recruits natural killer T (NKT) cells to accumulate in the liver and ultimately inhibits tumor growth. However, secondary BAs inhibit CXCL16 and favor tumor growth (104). The reduction of secondary BAs in the hepatocellular carcinoma process and the relative increase of primary BAs in the hepatocellular carcinoma process promote the accumulation of secondary BAs, which favors the aggregation of NKT cells in the liver, and the protective mechanism does not appear to be involved in the development of hepatocellular carcinoma.

The specific molecular mechanisms of BAs on hepatocellular carcinoma have also been explored. The relationship between TCA and the development of hepatocellular carcinoma is well established. Data in the clinic suggest that serum TCA is generally elevated in HCC patients compared to healthy controls. High serum TCA is associated with more M2-polarised M2-like tumor-associated macrophages (TAM) in clinical samples of HCC. In addition, Sirtuin 5 (SIRT5) was downregulated in human primary HCC samples (105). In Sirt5-deficient mice, SIRT5 synergizes with the oncogene via degree succinylation to increase BA production biosynthesis in hepatocyte peroxisomes (89). Thus, SIRT5 deficiency in hepatocellular carcinoma leads to altered BA biosynthesis in hepatocytes, and elevated BAs (including TCA) in turn activate FXR (Nr1h4) and skew macrophage polarization to produce an immunosuppressive tumor microenvironment (TME) that is conducive to tumor-initiating cell (TIC) expansion and tumor growth.

### MASLD/MASH

#### Homeostatic changes in BAs in MASLD/MASH

It is undeniable that MASLD/MASH leads to changes in BA homeostasis in the body (106). In animal experiments with HFC-induced MASH, elevated levels of hydrophobic BAs (DCA, CDCA, and CA) were observed (107–109). In patients with MASH/MASLD, primary BAs and the conjugated forms are markedly elevated. A study by Puri et al. (110) demonstrated that NASH patients exhibit elevated total primary BAs alongside reduced secondary BAs. A multicenter investigation by Lyu et al. (199) confirmed significantly higher serum levels of TBA, CA, CDCA, and UDCA in MASLD patients compared to healthy controls. Basuni et al. (111) further observed elevated CA, CDCA, and its glycine/taurine conjugates (GCA, GCDCA, TCA, and TCDCA) in both MASLD and NASH groups, with GCA and TCA significantly higher in NASH than MASLD, suggesting an association with inflammatory progression. Notably, tauro-conjugated BAs were enriched in MASH. Additionally, Hirata et al. (112) observed strong positive correlations between TCDCA and GCDCA with AST levels. The findings from Basuni et al. (111) indicated TCA as a significant predictor of active inflammation (OR = 1.94). Regarding fibrosis, CA (OR = 2.05), CDCA (OR = 1.58), GCA (OR = 1.92), and DCA (OR = 2.06) were significant predictors of fibrosis. Elevated bile salt levels increased the likelihood of *F* ≥ 2 fibrosis (*p* = 0.007), whereas an elevated secondary/primary bile acid ratio reduced fibrosis risk (OR = 0.57) (110).

#### BAs signaling as a therapeutic target in MASH

Regulatory therapies targeting the alternative synthesis pathway of BAs for the treatment of MASLD and MASH represent current research priorities (113). CYP7B1 (cytochrome P450 family 7 subfamily B member 1) serves as a pivotal enzyme within the alternative BA synthesis pathway (114). Research by Kakiyama et al. (115) demonstrated that liver-specific transgenic expression of CYP7B1 completely blocked Western diet-induced hepatotoxicity, providing proof-of-concept for targeting this alternative pathway. Genetic studies indicate that ALDH2 deficiency inhibits FXR/SHP signaling, downregulates CYP7B1 expression, and suppresses the alternative BA synthesis pathway, thereby exacerbating hepatic steatosis, inflammation, and fibrosis (116). Conversely, the natural compound celastrol activates the FXR/LXR signaling pathway, upregulates CYP7B1 expression, corrects the imbalance in the 12-OH/non-12-OH BAs ratio, restores the *de novo* BA synthesis pathway, and improves the MASH phenotype (117). Interestingly, HDCA levels are significantly reduced in patients with MAFLD (referred to as NAFLD in earlier publications) and negatively correlate with disease severity (118). Conversely, HDCA supplementation ameliorates MAFLD phenotypes by inhibiting intestinal FXR signaling, remodeling the microbiota, and ultimately upregulating hepatic the peroxisome proliferator-activated receptor alpha (PPARα) and CYP7B1 (118). Thus, these studies collectively suggest that targeting the alternative BA synthesis pathway holds therapeutic potential. However, clinical trials remain lacking to formally validate this approach.

Modulation of the gut microbiota is also a key focus in treating MASH/MASLD (119). HDCA therapy increases the abundance of probiotic species such as *Bacteroides distasonis*, which enhances lipid metabolism through fatty acid-mediated activation of PPARα pathway in the liver (118). Furthermore, the novel microbially derived bile acid 3-sucCA, produced by *Bacteroides uniformis*, alleviates MASH by promoting *Akkermansia muciniphila* growth (120), revealing a novel mechanism of gut microbiota-BA interaction.

#### BAs mediate intestinal flora that exacerbate liver disease

Recent studies have suggested that disruption of the intestinal epithelial barrier and the intestinal vascular barrier is thought to be an early event in the pathogenesis of MASH (121). The further progression of MASH leads to hepatic fibrosis, cirrhosis, and hepatocellular carcinomas (122), so that all of these liver diseases seem to be accompanied by a disruption of the intestinal barrier in an ongoing vicious circle. BA disorders *in vivo* have been thought to indirectly disrupt the intestinal barrier by affecting disorders of the bacterial flora. However, it has recently been found that coupled BAs protect the intestinal epithelial barrier from *in vitro* damage by sequestering unbound BAs in micelles. Therefore, changes in intestinal bacteria (changes in BSH activity) increase the abundance of bound BAs, affecting small intestinal barrier function and the development of hepatic inflammation in vivo (123). Prolonged alcohol citation leads to an increase in BSH-containing flora and the level of unconjugated BAs and a relative decrease in the percentage of conjugated BAs in the ileum of mice, which may lead to intestinal barrier damage and further induction of alcoholic hepatitis (124).

In addition to this, the reduced abundance of conjugated BAs in the intestine promotes BA synthesis in the liver and further exacerbates liver injury. Bile flow was higher in germ-free mice than in conventional mice. In addition, changes in the gut microbiota were found to directly affect Asbt expression controlled by the transcription factor Gata4, increasing absorption and decreasing BA synthesis (125).

Many attempted studies have found that supplementation with BAs or their analogs have found that their therapeutic effects seem to involve changes that can affect the intestinal flora. Intervention with the BA derivative Obeticholic acid (OCA) was able to enrich specific gut microbes (*Akkermansia muciniphila*, Bifidobacterium spp., Bacteroides spp., Alistipes spp., Lactobacillus spp., *Streptococcus thermophilus*, and *Parasutterella excrementihominis*) and regulate host BAs (126). HDCA supplementation (118) increases the abundance of norank_f_Muribaculaceae (127) in the gut microbiota (128). Supplementation with DCA modulated the composition of the gut flora, characterized by a significant increase in the genera Akkermansia and Butyricoccus and a significant decrease in the genera Turacibacter and Lactobacillus. The most prominent effect was observed in *A. muciniphila* (129). In mice supplemented with UDCA it was found to have no effect on the normal group of mice, but regulated UDCA in HFHC mice to decrease or recover genera Bacteroides, Parabacteroides, Roseburia, and Intestininomas and, at the same time, elevate and recover norank_f_ Muribaculaceae, Parasutterella (130). The distribution of intestinal flora differed among responders, non-responders, and poor prognosticators of primary biliary cholangitis treated with UDCA. The abundance of bsh gene-carrying gates was lower in poor prognosticators than in responders (131). In HFHC-fed mice supplemented with TUDCA the colonies were restored to the normal group, but the effect of TUDCA feeding on the flora in the normal group was not examined (132).

#### Regulation of BAs homeostasis is a key approach to treating liver disease

An interesting recent study found that intestinal inflammation and barrier damage found in Dextran sulfate sodium (DSS)-induced colitis ameliorated acute cholestatic liver injury and led to reduced hepatic fibrosis (133) in a model of chronic colitis. It is possible that colitis-induced hepatocyte NF-κB activation leads to significant inhibition of the hepatic transcriptional program for BA synthesis, uptake, and secretion, which in turn leads to hepatic BA accumulation and reduced systemic BA levels. Studies have once again demonstrated the reliability of lowering *in vivo* bile depot levels for the treatment of liver disease. Currently, there are several main therapeutic approaches to regulate BA homeostasis in the treatment of liver disease: (i) BAs and their derivatives, such as HDCA, UCDA; (ii) BA receptor agonists or antagonists, such as FXR agonists and TRG5 agonists; (iii) lowering of total BA pools in the body, such as BA sequestrants, FGF analogues, ASBT, and HMGCR inhibitors; and (iv) regulation of intestinal flora to influence BA reabsorption, e.g., probiotics (see Tables 1–3 below for details). In fact, it has been found that many drugs can treat several different liver diseases at the same time, such as the FXR agonist obeticholic acid, BA chelators, and UCDA, which reinforces the fact that the imbalance of BAs is a common etiology of these liver diseases, and even more so, a common target for the treatment of these liver diseases.

**Table 1:** Regulation of BAs for the treatment of MASLD/MASH.

Drug		Targets involved	Clinical trial phase	Key efficacy outcomes	Safety/adverse events	Regulatory status	Reference
BAs and their derivatives	norUDCA	Inhibition of intestinal FXR, upregulation of hepatic CYP7B1, PPARα and FXR	Phase 3	57% of patients achieved fibrosis improvement, and the ALT normalization rate reached 89%	Gastrointestinal events	Not approved	(139)
	Obeticholic acid	Increased expression of FXR and FGF19, upregulation of Cyp7b1	Phase 3	Improved liver histology and liver enzymes	Dyslipidemia, pruritus, cholelithiasis, and liver toxicity risk	Terminated	(140, 141)
	Volixibat	Suppression of ABST	Phase 2	No therapeutic effect	Diarrhea	Terminated	(142–144)
FGF19 analogue	Aldafermin	Inhibition of FXR receptors	Phase 2b	Improved non-invasive fibrosis markers such as enhanced hepatic fibrosis score (ELF), APRI, FIB-4, and blood lipid ratio (TG/HDL)	Diarrhea, feeling sick, abdominal pain.	Not approved	(145)
FGF21 analogue	Pegbelfermin	Reduced relative abundance of genes encoding cholylglycine hydrolases	Phase 2	Improved MASH and biomarkers including adiponectin and PRO-C3	Diarrhea	Terminated	(146–148)
HMGCR inhibitors	Atorvastatin	Suppression of adipogenic genes and enhancement of Cyp7a1 hepatic expression	Phase 4	In a large cohort study, statin use was associated with significantly lower risks of liver-related events and liver stiffness progression	——		(146, 149)
Natural ingredients	berberine	Up-regulation of Cyp27a1, Cyp2e1, Cyp7b1, Cyp8b1, Ntcp, Abcc3, activation of FXR	Phase 3	No significant reduction in visceral adipose tissue area or liver fat content compared to placebo	Generally tolerable	Not approved	(150–153)
	HTD1801 (berberine ursodeoxycholate)	Activate FXR/TGR5 signal; Intestinal flora remodeling; Optimization of BAs composition	Phase 3	Significantly reduced ALT, AST, and GGT levels; reduction in liver fat content of ≥30%	Mild-to-moderate gastrointestinal events	FDA Fast Track designation for NASH/MASH	(154)
	*Portulaca oleracea* (common purslane)	Up-regulation of CYP7A1 mRNA and down-regulation of FXR mRNA	Phase 2/3	Liver stiffness decreased significantly; Significant reductions in ALT, AST, and GGT	Excellent safety profile	Not approved	(155, 156)
	curcumin	Increased faecal BSH activity and up-regulation of TGR5 mRNA expression levels increased serum GLP-1	Phase 3	Significantly improved hepatic steatosis and liver stiffness	GRAS (Generally Recognized as Safe) status by FDA	Not approved	(156–158)

HDCA, hyodeoxycholic acid; FXR, Farnesoid X receptor; CYP7A1, Cholesterol 7α-hydroxylase; PPARα, Peroxisome proliferator-activated receptor alpha; FGF15/19, Fibroblast growth factor 15/19; FGF21, fibroblast growth factor 21; UDCA, ursodeoxycholic acid; Cyp27a1, Mitochondrial sterol 27-hydroxylase; Cyp2e1, Cytochrome P450 2E1; Cyp7b1, Cytochrome P450 Family 7 Subfamily B Member 1; Cyp8b1, Cytochrome P450 Family 8 Subfamily B Member 1; NTCP, Sodium/taurocholic acid cotransport polypeptide; Abcc3, ATP binding cassette subfamily C member 3; BSH, Bile salt hydrolase; TGR5, Takeda G protein-coupled receptor 5; GLP-1,glucagon-like peptide-1; FDA, U.S. Food and Drug Administration; EMA, European Medicines Agency.

**Table 2:** Drugs for regulating BAs in the treatment of hepatocellular carcinoma.

Intervention drugs		Design targets	Mechanisms and advances in the regulation of BA homeostasis	Reference
FGF19 analogue	FGF19-M52	Inhibition of CYP7A1	Reduces BA pool in the body by inhibiting hepatic BA synthesis-increased transaminases in clinical studies, common adverse effect of diarrhea alone	(159–161)
BAs transporter protein inhibitor	SC-435, A4250	ASBT	ASBT inhibition increases colonic BA accumulation, reduces BA pool, and regulates BA metabolism Altered BA homeostasis and significantly altered biliary tract BA composition	(159, 162)
Probiotics	VSL#3	Regulation of intestinal flora, FGF15, FXR pathway	Promotes ileal BA dissociation and induces hepatic BA de novo synthesis by down-regulating the enterohepatic FXR-FGF15 axis Promotes BA excretion and improves BA metabolism	(163–165)
BAs and their analogues	OCA	FXR, CYP7A1	There is a lack of clinical data on OCA for liver cancer, but several clinical trials have demonstrated the efficacy of OCA in the treatment of chronic liver disease. Combines OCA and nanomaterials to enhance FXR agonism and effectively regulate BAs	(159, 166)
	UDCA	E2F-1/Mdm-2/p53 Apoptosis pathway, ROS	Interferes with the E2F-1/Mdm-2/p53 apoptotic pathway, leading to subsequent nuclear translocation of the BA receptor complex and reduced apoptosis Stimulates biliary tract damage, alters the BA pool, and reduces fecal concentrations of secondary BAs and deoxycholic acid Clinical studies have shown that the 5-year cumulative incidence of HCC in patients treated with UDCA is 17.9%, which is significantly lower than that in patients not treated with UDCA	(163, 167, 168)
Non-steroidal FXR agonists	px20606, px-102, px-104	HRG, FGF19, inhibition of CYP7A1, NDRG2	Prevents BA carrier overload Reduces total BAs and regulates BA balance. Elevated HRG levels in plasma receiving only PX20606 in clinical studies	(169–171)
Secondary BAs	DCA, LU	TGR5, antagonism of NF-κB, AKT, ERK pathway	Maintains BA levels and regulates BA-mediated signaling pathways Stimulates the traditional BA synthesis pathway and inhibits alternative pathways	(172–174)
LCA-related synthetic compounds	LCA-acetate, LCA-propionic acid	VDR, SHP, CYP7A1	Increased BA synthesis and mediated BA metabolism VDR inhibits SHP, increased expression and activity of CYP7A1, and increased BA pool size and fecal BA excretion	(172, 175, 176)

FGF15/19, Fibroblast growth factor 15/19; CYP7A1, Cholesterol 7α-hydroxylase; ASBT, Apical sodium-dependent BAs transport protein; FXR, Farnesoid X receptor; OCA, Obeticholic acid; UDCA, Ursodeoxycholic acid; E2F1, E2F transcription factor 1; Mmousedouble minute 2;p53, Cellular tumor antigen p53; ROS, Reactive oxygen species; HRG, histidine-rich glycoprotein; NDRG2, N-Myc downstream regulated gene 2; DCA, Deoxycholic acid; TGR5, Takeda G protein-coupled receptor 5; NF-κB,nuclear factor kappa B; AKT, protein kinase B; ERK, Extracellular regulated protein kinases; LCA, Lithocholic acid; VDR; Vitamin D receptor; SHP, Small heterodimer partner.

**Table 3:** Drugs that regulate BAs for the treatment of cholestasis.

Intervention drugs		Mechanisms of BAs regulation	Clinical trial phase	Key efficacy outcomes	Safety/adverse events	Regulatory status	Reference
BAs and their analogues	UDCA	Relative replacement of lipophilic, deterge not approved	Phase 4	Significantly improves biochemical markers ALP and Bilirubin	Well-tolerated.	FDA/EMA: Approved for PBC.	(177–181)
	norUDCA		Phase 3	57% of patients achieved fibrosis improvement; ALT normalization rate reached 89% (vs. 76% placebo).	Well-tolerated. No serious AEs reported in the Phase 3 trial.	Not approved	(182, 183)
FXR receptor agonists	OCA	OCA increases the transport of BAs from blood to bile.	Phase 3	Significantly improves biochemical markers ALP and Bilirubin	Pruritus	FDA: Approved (2016) for PBC. EMA: Withdrawn conditional authorization (2024).	(183–187)
	Cilofexor	Activation of FXR receptors to reduce BAs production	Phase 3	Phase 3 PRIMIS study (NCT03890120) is ongoing. Phase 2 studies showed significant improvements in cholestasis markers (ALP and GGT).	Generally well-tolerated in Phase 2; Phase 3 results pending.	Not approved.	(188–190)
Ileal BAs transporter protein inhibitor	GSK2330672	Interruption of enterohepatic circulation of BAs	Phase 3	57% itch reduction and 50% BA reduction.	Gastrointestinal events	Under Review (for PBC pruritus); Orphan Drug Designation granted by both FDA and EMA.	(191–193)
Ileal BAs transporter protein inhibitor	Odevixibat	Interruption of enterohepatic circulation of BAs	Phase 3	Significantly improved pruritus and reduced serum BA (33% responders vs. 0% placebo).	Gastrointe-stinal events	FDA/EMA: Approved for pruritus in PFIC patients ≥6 months.	(194–196)
Ileal BAs transporter protein inhibitor	Maralixibat	Interruption of enterohepatic circulation of BAs	Phase 3	Significantly improved pruritus and reduced serum BA.	Gastrointe-stinal events	FDA/EMA: Approved for cholestatic pruritus in ALGS patients ≥1 year; also approved for PFIC.	(197, 198)

UDCA, ursodeoxycholic acid; norUDCA, norursodeoxycholic acid; OCA, obeticholic acid; FXR, farnesoid X receptor; PSC, primary sclerosing cholangitis; PFIC, progressive familial intrahepatic cholestasis; PBC, primary biliary cirrhosis. FDA, U.S. Food and Drug Administration; EMA, European Medicines Agency.

However, most of the drugs targeting the regulation of BAs for the treatment of these liver diseases regulate the total BA levels as a whole, and there are very few drugs targeting the specific changes in BAs in different liver diseases. On the one hand, it may be that the changes in BAs in different liver diseases are still controversial but, on the other hand, it may be difficult to grasp the changes as they may be dynamic in development.

## Conclusion and outlook

The liver, as the largest gland in the human body, plays a crucial role in metabolism, detoxification, and immunity. However, existing therapies for liver diseases such as cirrhosis, HCC, and MASLD commonly show limited and unsatisfactory therapeutical effects in clinic (134), underscoring the urgent need to explore novel therapeutic targets. BA accumulation is one of the earliest identified pathogenic mechanisms. Recent studies reveal its effects extend beyond cytotoxicity to inflammation, immune regulation, and metabolic disorders (135). This review summarizes the toxic effects of BAs on organelles and their compositional shifts across different liver diseases.

Abnormal BA profiles hold promise as novel “monitors” for liver diseases, enabling subtype differentiation and progression prediction. However, current applications remain limited to measuring total BA concentrations, lacking in-depth exploration of specific BA associations with disease. Future efforts should establish large-scale longitudinal BA metabolomics cohorts to collect serial samples across disease stages. Combining machine learning to establish specific BA thresholds for distinguishing subtypes and predicting risk could transform BAs from auxiliary indicators into true diagnostic tools.

Currently, drugs targeting BA homeostasis (e.g., FXR and ASBT inhibitors) show efficacy in experimental animal models but often face clinical trial setbacks due to side effects such as pruritus and dyslipidemia. This underscores the necessity for human validation of target functions, for instance, the potential role of NTCP in hepatic stellate cell (HSC) activation requires single-cell sequencing verification using human cirrhotic liver samples. Concurrently, deepening research on the gut microbiota-BA axis has spurred probiotic strategies, though single-strain interventions carry risks. Future efforts should pivot toward multi-omics-guided precision microbiome-BA therapies. These should design synergistic microbial communities or engineered bacteria based on individual metagenomic and BA profile characteristics to achieve precise regulation of BA homeostasis. It is also urgent to develop more reliable candidate drugs for the treatment of liver diseases with higher safety. In recent years, accumulating evidence has suggested that natural agents from foods or herbal medicines are trustworthy resources for discovering candidate drugs for the management of various diseases, and previous literature also indicated that abundant natural agents from foods or herbal medicines could be considered as hepatoprotective supplements (136–138). Consequently, more work could be devoted to the study of hepatoprotective agents via regulation of BA homeostasis derived from foods, herbal medicines, and their formulas, which would be beneficial for the development of novel drugs and the treatment of liver diseases in the future.
